# Association of high risk of liver fibrosis in patients with serious outcomes of adverse drug reactions

**DOI:** 10.1007/s00228-026-04057-z

**Published:** 2026-04-23

**Authors:** Joana Henze, Harald Dormann, Carolin V. Schneider, Corinna Meeßen, Anja Knüppel-Ruppert, Andrea Kriegisch-Stumpf, Matthias Schwab, Julia C. Stingl, Katja S. Just

**Affiliations:** 1 Institute of Clinical Pharmacology, University Hospital RWTH Aachen, Wendlingweg 2, Aachen, D-52074 Germany; 2 Emergency Department, Klinikum Fürth, Fürth, Germany; 3 Department of Medicine III, University Hospital RWTH Aachen, Aachen, Germany; 4 Central Emergency Department, Hospital Ingolstadt, Ingolstadt, Germany; 5 Dr. Margarete Fischer-Bosch-Institute of Clinical Pharmacology, Stuttgart, Germany; 6 Department of Clinical Pharmacology, and Pharmacy and Biochemistry, University Tübingen, Tübingen, Germany; 7 Department of Clinical Pharmacology and Pharmacoepidemiology, Heidelberg University Hospital, Heidelberg, Germany

**Keywords:** Adverse drug reaction, Liver fibrosis, Metabolic dysfunction–associated steatotic liver disease, Drug treatment

## Abstract

**Purpose:**

Metabolic dysfunction–associated steatotic liver disease (MASLD) as the main cause of chronic liver disease worldwide is associated with increased mortality. Especially in patients with fibrosis, an increased risk of adverse drug reactions (ADRs) might be present. Therefore, the impact of high-risk for liver fibrosis on the outcome of ADRs was analyzed.

**Methods:**

Patients of a multicenter cohort study in maximum care emergency departments (ED) with admissions due to ADRs with available FIB-4 score parameters were analyzed. The cohort was divided into patients with low (FIB-4 < 2.67) and high (FIB-4 ≥ 2.67) liver fibrosis risk. Triage at ED admission, ADR seriousness criteria, discharge status, and length of hospital stay were used as outcomes. Logistic and linear regression analyses were applied.

**Results:**

Among the 1087 patients, 407 had a high risk of fibrosis (37.4%). High risk of fibrosis was associated with serious ADRs (OR 2.42 (95% CI 1.57–3.73)), a poor discharge status including deaths (3.51 (2.20–5.60)), and a longer stay in the hospital (2.02 (1.00–3.05)), while urgent triage at ED admission was not significantly associated with high fibrosis risk (0.95 (0.71–1.28)).

**Conclusion:**

In patients at risk of MASLD, careful risk assessment is essential when prescribing drugs, as serious ADRs may occur unnoticed in the presence of liver fibrosis. Even simple non-invasive tests like the FIB-4 score may provide valuable prognostic hints in the acute event of an ADR.

**Supplementary Information:**

The online version contains supplementary material available at 10.1007/s00228-026-04057-z.

## Introduction

Adverse drug reactions (ADRs) are a common reason for visits to the emergency department and subsequent hospitalization [1–3]. It is plausible that several vulnerability factors may be associated with the occurrence of ADRs and a severe or serious outcome [4]. The liver plays a central role in detoxification such as drug metabolism. Hence, organ damage to the liver, reducing liver function is expected to decrease hepatic clearance, thereby increasing ADR risk [5]. While this was shown for liver cirrhosis, it is so far unclear at what disease state, the hepatic clearance decreases along with a higher ADR risk or respective a poorer ADR outcome.

Metabolic dysfunction–associated steatotic liver disease (MASLD), which is the new nomenclature replacing the old disease term nonalcoholic fatty liver disease (NAFLD), is a common condition in Western countries [6]. According to the definition, a cardiometabolic risk factor must exist in addition to liver steatosis to diagnose MASLD [7]. Despite the introduction of new diagnostic criteria, evidence indicates that most patients previously diagnosed with NAFLD still meet these criteria. Therefore, findings conducted under the NAFLD definition remain applicable within the redefined MASLD classification [8, 9]. Thus, MASLD continues to be the leading cause of chronic liver disease worldwide [6, 10, 11], which has increasing prevalence and relevance to society every year [6, 12, 13]. Patients with MASLD are often multi-morbid including diseases of the metabolic syndrome [14, 15], which is commonly associated with polypharmacy [16]. Furthermore, studies indicate altered pharmacokinetics in MASLD patients [17]. These, in combination with a potentially impaired hepatic function, increase the risk of serious ADRs [18–20].

During the course of MASLD, liver fibrosis and subsequently cirrhosis can occur [15]. To assess the risk of this progression, international guidelines recommend the non-invasive FIB-4 score, which classifies patients into high- and low-risk fibrosis groups based on four parameters [21]. As these advanced stages of the disease in particular are associated with increased overall mortality [22], the question arises as to whether fibrosis and cirrhosis of the liver could have an influence on ADRs and their outcome. Although in general, hepatic drug metabolism often persists until cirrhosis [23, 24], a previously damaged liver could have relevant effects on the frequency, seriousness and outcome of ADRs. The aim of this study is to analyze the association of high-risk liver fibrosis in the context of MASLD on ADRs in an emergency department population, its seriousness and outcome.

## Materials and methods

### Study design and data collection

We used a dataset of the multicenter, prospective ADRED study, that collected cases of ADRs in six emergency departments (EDs) from the beginning of September 2015 to the end of December 2021 in tertiary care hospitals in Germany. These included the Department of Clinical Acute and Emergency Medicine at Bonn University Hospital, the Internal Medicine Emergency Department at Ulm University Hospital, the Central Emergency Department at Fürth Hospital, the Department of Clinical Acute and Emergency Medicine at Robert Bosch Hospital in Stuttgart, the Clinic for Acute and Emergency Medicine at Ingolstadt Hospital and the Central Emergency Department at Augsburg University Hospital. During the study period, all patient files were systematically checked by trained study personnel to determine whether their symptoms could have a causal relationship with an ADR. In addition, information provided by treating physicians was checked. The systematic causality assessment was based on the WHO-UMC system [25], which enabled a structured assessment of the causality of the symptoms and their association with an ADR. All cases with ADRs according to the causality criteria “possible”, “probable” and “certain” were pseudonymized and included in the study. All patients were of legal age and consented to study inclusion after being informed. If they were unable to give consent due to the seriousness of the ADR, participation in the study was approved by the legal guardian or the patients were included anonymously. Further details on study enrollment are published elsewhere [1, 26]. The study was approved by the responsible ethics committee at the University of Bonn (202/15).

### Study population and variable specification

We included all inpatient cases in our analysis with complete information on variables enabling FIB-4 score calculation, i.e. age, alanine aminotransferase (ALT), aspartate aminotransferase (AST), and platelet count.

The FIB-4 scores were calculated using the formula FIB-4 = age (years) x AST (U/l) / (thrombocytes (10^9^/l) x (ALT (U/l))^1/2^) [27]. Based on calculated FIB-4 scores, patients were divided into two groups with a low or intermediate and a high risk of advanced liver fibrosis. The established cut-off ≥ 2.67 was used, which predicts a high risk of advanced fibrosis with an 80% positive predictive value [28].

### Covariables

Other variables recorded were age and sex, as well as the number of known illnesses and taken drugs. We evaluated the cohort for known diseases of interest as depicted by ICD-10 codes documented. We analyzed the prevalence of diabetes (E10-E14), essential hypertension (I10), dyslipidemia (E78), and liver diseases (R17, B15-B19, K70-K76). In addition, the patients were asked about their alcohol consumption, with continuous and episodic abuse being summarized as alcohol abuse. We analyzed ADR symptoms as coded by the Medical Dictionary for Regulatory Activities (MedDRA) terminology on a preferred term level. We searched for the following ADR symptoms, as we expected them to be potentially liver-specific: nausea, vomiting, weight loss, stool discoloration, ascites, jaundice, urinary abnormalities, upper abdominal pain and distended abdomen, enlarged waist circumference, and pruritus excluding allergic pruritus. Triaging in the ED was carried out by trained staff according to the Manchester Triage System (MTS) or the Emergency Severity Index (ESI) in red, orange, yellow, green and blue. The presentation with a liver-specific admission diagnosis was also considered. We defined an acute ED admission due to a liver disease as K71-K76 but excluded chronic hepatitis (K73).

### Outcomes

We used different outcome measurements to follow the hypothesis of a poorer ADR outcome with higher fibrosis risk. The triage was used to assess the urgency of the ADR by using a binary outcome, combining cases classified as red (immediate) or orange (very urgent) and testing these versus others. A serious ADR was defined using ADR seriousness criteria as a stay in the intensive care unit (ICU), either by immediate admission to the ICU or a necessary admission during the course of normal inpatient treatment, a life-threatening ADR, and death due to the ADR. The discharge status of the patients was also assessed. A distinction was made between a poor discharge status defined as permanent or fatal damage. Finally, the length of stay (LOS) in hospital was analyzed as a continuous outcome parameter.

### Statistical analysis

Characteristics and ADR symptoms on ED admission of patient groups with high versus low or intermediate fibrosis risk according to FIB-4 scores were compared. Data were expressed as absolute and relative frequency for categorical, and median with interquartile range (IQR) for continuous variables. Categorical variables were compared using chi-squared test including, were possible, a Bonferroni adjusted Z-test, and continuous variables were compared using Mann-Whitney U-test. In addition, a descriptive analysis was performed of the drug intake documented at the day of the ADR. We tested whether serious ADRs were associated with liver fibrosis as expressed by high fibrosis-risk scores. To this end, we tested a high fibrosis-risk according to FIB-4 compared to low or intermediate risks (low fibrosis-risk). We used binary logistic regression adjusting for variables that we expected to be potential confounders. To adjust associations of fibrosis according to FIB-4, we used age, male sex, and number of documented taken drugs. We did comparable analyses with discharge status for having a poor outcome and urgent triage at ED admission, and linear regression for LOS in days. Results are shown by odds ratios (OR) with associated 95% confidence interval (CI) for binary logistic regression analysis and unstandardized b-coefficients with 95% CI for linear regression analysis. A p-value < 0.05 was considered statistically significant. All statistical analyses were performed with the program IBM SPSS Statistics 29.0.1.1.

## Results

In total, *N* = 1087 patient cases with *n* = 407 patients (37.4%) with high and 680 (62.6%) with low or intermediate liver fibrosis-risk were included in the study. The characteristics of the study population are shown in Table 1. The median FIB-4 of patients at low risk of liver fibrosis was 1.31 (IQR 0.85;1.86) and 5.12 (IQR 3.41;10.54) in patients with high fibrosis-risk. All single parameters that are part of FIB-4 calculation, age, thrombocyte count, and liver enzymes ALT and AST differed significantly between the two groups.

**Table 1:** Patient characteristics of the study cohort (*n* = 1087) stratified by patients with and without liver fibrosis risk according to FIB-4 score

	Missings, *n* (%)	With low or intermediate liver fibrosis risk, *n* = 680 (62.6%)	With high liver fibrosis risk, *n* = 407 (37.4%)	*p*-value
**Fibrosis- (FIB-4) score calculation**				
FIB-4 score, median (IQR)	-	1.31 (0.85; 1.86)	5.12 (3.41;10.54)	**< 0.001**
Age (years), median (IQR)	-	70 (57;79)	77 (66;83)	**< 0.001**
Thrombocytes (10^9^/l), median (IQR)	-	263 (208;336)	113 (61;174)	**< 0.001**
ALT (U/l), median (IQR)	-	19 (14;29)	23 (15;44)	**< 0.001**
AST (U/l), median (IQR)	-	21 (16;28)	36 (24;63)	**< 0.001**
**Other clinical characteristics**				
Male sex, n (%)	-	348 (51.2)	229 (56.3)	0.104
BMI (kg/m²), median (IQR)	451 (41.5)	26.4 (23.0;30.1)	25.4 (22.8;29.4)	0.141
Diabetes, n (%)	29 (2.7)	160 (24.4)	89 (22.1)	0.383
Dyslipidemia, n (%)	29 (2.7)	162 (23.8)	98 (24.1)	0.924
Essential hypertension, n (%)	29 (2.7)	352 (51.8)	233 (57.3)	0.079
Number of previous illnesses, median (IQR)	29 (2.7)	8 (5;13)	11 (7;15)	**< 0.001**
Number of prescribed drugs, median (IQR)		7 (4;11)	8 (5;11)	**0.018**
Diagnosed liver disease, n (%)	29 (2.7)	39 (6.0)	56 (13.9)	**< 0.001**
Diagnosed liver disease, excluding alcoholic liver disease, n (%)	29 (2.7)	36 (5.5)	46 (11.4)	**< 0.001**
Viral hepatitis (B15-B19), n (%)	29 (2.7)	1 (0.2)	4 (1.0)	0.053
Alcoholic liver disease (K70), n (%)	29 (2.7)	7 (1.1)	20 (5.0)	**< 0.001**
Toxic liver disease (K71), n (%)	29 (2.7)	1 (0.2)	1 (0.2)	0.728
Liver failure (K72), n (%)	29 (2.7)	2 (0.3)	10 (2.5)	**0.001**
Fibrosis and cirrhosis of the liver (K74), n (%)	29 (2.7)	8 (1.2)	23 (5.7)	**< 0.001**
Inflammatory liver disease (K75), n (%)	29 (2.7)	1 (0.2)	3 (0.7)	0.128
Other diseases of the liver (K76), n (%)	29 (2.7)	26 (4.0)	12 (3.0)	0.4
Fatty liver (K76.0), n (%)	29 (2.7)	17 (2.6)	7 (1.7)	0.362
Hyperbilirubinemia (R17), n (%)	29 (2.7)	0 (0.0)	0 (0.0)	
Chronic hepatitis (K73), n (%)	29 (2.7)	0 (0.0)	0 (0.0)	
**Alcohol abuse**, n (%)	622 (57.2)	93 (31.4)	67 (39.6)	0.073
None, n (%)		203 (68.6)	102 (60.4)	> 0.05
Continuous, n (%)		32 (10.8)	34 (20.1)	**< 0.05**
Episodic, n (%)		61 (20.6)	33 (19.5)	> 0.05

High-fibrosis risk defined as FIB-4 score ≥ 2.67; *IQR,* interquartile range; *ALT,* alanine aminotransferase; *AST,* aspartate aminotransferase; *BMI,* body mass index; *PT,* prothrombin time; Diagnosed liver disease defined as the presence of one or more of the listed liver diseases (R17, B15-B19, K70-K76)

Significant findings in **bold** text

High fibrosis-risk patients had significantly more pre-existing conditions (11 (IQR 7;15)) with concordant higher number of documented drug intake (8 (5;11)) compared to low and intermediate risk (8 (5;13) diseases with 7 (4;11) drugs). Drugs documented for the population showed no distinctive differences between the two groups. A descriptive overview can be found in Supplement 2. The high fibrosis-risk group had significantly more often previously diagnosed liver disease (*n* = 56 (13.9%)) than the low fibrosis-risk group (*n* = 39 (6.0%)). Among the pre-diagnosed liver diseases, fibrosis and cirrhosis of the liver (*n* = 23 (5.7%)), alcoholic liver disease (*n* = 20 (5.0%)), and liver failure (*n* = 10 (2.5%)) were more often documented in the group of high fibrosis-risk compared to low fibrosis-risk (with *n* = 8 (1.2%), *n* = 7 (1.1%), and *n* = 2 (0.3%)).

Many ADR symptoms, that we defined as potentially liver-specific were not significantly different distributed between low and high fibrosis-risk groups (Table 2). Exceptions were jaundice and ascites that were found significantly more often in patients with high fibrosis-risk, although only small numbers were observed (*n* = 4 (1.0%) and *n* = 6 (1.5%) in high risk versus *n* = 1 (0.1%) and *n* = 1 (0.1%) in low fibrosis-risk group). Meanwhile, liver-specific admission diagnoses did not differ between groups and were only observed in a small number of patients. The number of patients being triaged as immediate or very urgent cases on ED admission did not differ significantly. However, serious ADRs and a poor discharge status were found significantly more often in patients with high fibrosis-risk (*n* = 56 (13.8%) and *n* = 62 (15.7%)) compared to without (*n* = 43 (6.3%) and *n* = 30 (4.6%)) with a higher occurrence of deaths (Supplement 1). Further details on triage at admission, ADR seriousness, and subsequent discharge status are shown in Supplement 1. Patients with high fibrosis-risk stayed significantly longer in the hospital (median 7 (4; 12)) compared to low fibrosis-risk (6 (3; 10)).

**Table 2:** Clinical presentation of the study cohort (*n* = 1087) stratified by patients with and without liver fibrosis risk according to FIB-4 score at emergency department admission

	Missings, *n* (%)	With low or intermediate liver fibrosis risk, *n* = 680 (62.6%)	With high liver fibrosis risk, *n* = 407 (37.4%)	*p*-value
**Liver-specific symptoms of ADRs**				
Nausea, n (%)	-	98 (14.4)	54 (13.3)	0.599
Vomiting, n (%)	-	72 (10.6)	44 (10.8)	0.908
Decreased weight, n (%)	-	28 (4.1)	22 (5.4)	0.327
Discolored stool, n (%)	-	5 (0.7)	1 (0.2)	0.292
Pruritus, n (%)	-	10 (1.5)	3 (0.7)	0.282
Upper abdominal pain, n (%)	-	37 (5.4)	14 (3.4)	0.131
Abdominal distension, n (%)	-	4 (0.6)	4 (1.0)	0.461
Jaundice, n (%)	-	1 (0.1)	4 (1.0)	**0.049**
Enlarged waist circumference, n (%)	-	2 (0.3)	2 (0.5)	0.603
Ascites, n (%)	-	1 (0.1)	6 (1.5)	**0.008**
Abnormality of urine, n (%)	-	0 (0.0)	0 (0.0)	
**Liver-specific admission diagnosis**				
Toxic liver disease (K71), n (%)	-	1 (0.1)	1 (0.2)	0.713
Fibrosis and cirrhosis of the liver (K74), n (%)	-	0 (0.0)	2 (0.5)	0.067
Other diseases of the liver (K76), n (%)	-	1 (0.1)	2 (0.5)	0.295
Liver failure (K72), n (%)	-	0 (0.0)	0 (0.0)	-
Other inflammatory liver disease (K75), n (%)	-	0 (0.0)	0 (0.0)	-
**Clinical consequences of ADRs**				
Urgent triage red and orange, n (%)	13 (1.2)	161 (23.9)	95 (23.8)	0.987
Serious ADR, n (%)	-	43 (6.3)	56 (13.8)	**< 0.001**
Poor discharge status, n (%)	42 (3.9)	30 (4.6)	62 (15.7)	**< 0.001**
Length of stay, median (IQR)	-	6 (3;10)	7 (4;12)	**< 0.001**

High-fibrosis risk defined as FIB-4 score ≥ 2.67; *IQR,* interquartile range; *ADR,* adverse drug reaction; serious ADR defined as a stay in the intensive care unit, a life-threatening ADR and death; poor discharge status defined as permanent or fatal damage

Significant findings in **bold** text

We observed a higher chance for a serious ADR with high risk for liver fibrosis by OR 2.42 (95% CI 1.57–3.73) adjusted for age, sex, and number of prescribed drugs (Table 3). Likewise, a higher chance for a poor discharge status was seen for patients with high fibrosis risk (OR 3.51 (95% CI 2.20–5.60)) in logistic regression analysis. In both models, male sex was associated significantly with a poorer outcome with OR 1.73 (1.12–2.68) for serious ADRs and 2.33 (1.44–3.75) for poor discharge status. A high fibrosis-risk was significantly associated with a longer LOS by 2.02 (1.00 3.05) in linear regression analysis. The triage at ED admission, however, was not significantly associated with a high fibrosis risk.

**Table 3:** Regression analyses for associations of outcomes or adverse drug reactions (ADRs) with high liver fibrosis risk

	Urgent Triage, OR (95% CI)	Serious ADR, OR (95% CI)	Poor discharge status, OR (95% CI)	Length of stay, b-coefficient (95% CI)
High fibrosis risk	0.95 (0.71 – 1.28)	**2.42 (1.57 – 3.73)**	**3.51 (2.20 – 5.60)**	**2.02 (1.00 – 3.05)**
Age	1.00 (0.99 – 1.01)	1.00 (0.98 – 1.01)	1.01 (0.99 – 1.03)	0.01 (-0.03 – 0.04)
Male sex	**1.47 (1.11 – 1.96)**	**1.73 (1.12 – 2.68)**	**2.33 (1.44 – 3.75)**	0.10 (-0.87 – 1.07)
Number of prescribed drugs	1.03 (1.00 – 1.07)	0.98 (0.93 – 1.03)	1.00 (0.95 – 1.06)	0.22 (0.10 – 0.34)

*ADR,* adverse drug reaction; urgent triage defined as red (immediate) and orange (very urgent); serious ADR defined as a stay in the intensive care unit, a life-threatening ADR and death; poor discharge status defined as permanent or fatal damage; high-fibrosis risk defined as FIB-4 score ≥2.67; *OR,* Odds Ratio; *CI,* confidence interval

Significant findings in **bold** text

## Discussion

This study shows an association for ADRs with poorer outcomes in patients with high risk of liver fibrosis. This was observed for serious ADRs, poorer discharge status, and longer hospital LOS.

To the best of our knowledge, we show for the first time in this study an association of poorer outcomes after ADR among patients with a high liver fibrosis risk. While prevalence of MASLD as one important and frequent liver disease is expected to increase further in the next years [29], those patients are expected to be treated with a substantial number of drugs also due to a significant comorbidity [30]. Meanwhile, with higher drug intake, the risk for ADRs increases [31] exposing patients with liver diseases to a high risk for ADRs. These ADRs are often serious and with poorer outcomes. As the most common cause of chronic liver disease with a global prevalence of 30%, MASLD is a challenge for the healthcare system and will become increasingly relevant for patients and medical staff in the future [6, 10, 15]. Advanced stages of the disease with fibrosis and cirrhosis in particular are associated with increased overall mortality [22]. The LOS was also significantly longer, meaning that the treatment of these high-risk patients was associated with higher financial and personnel costs. This must be added to the already known economic consequences of the global epidemiological development of MASLD [6, 15]. Particularly striking in this study was the large proportion of patients with a high risk of liver fibrosis in contrast to patients with diagnosed fibrosis and cirrhosis of the liver. Other studies indicate that there could be a lot of asymptomatic and unrecognized patients with MASLD or metabolic dysfunction–associated steatohepatitis (MASH) and that a lot of patients with a high risk of liver disease are unaware of it [32, 33]. One of the reasons for this could be that liver-related complications often only occur in patients with cirrhosis as the overall mortality and the incidence of liver-specific complications increases parallel with the MASLD fibrosis stage [34]. However, our data now points to a need to recognize already patients with high-risk of liver fibrosis or suspected MASLD or MASH as high-risk patients for drug treatment. We used the FIB-4 score and the resulting fibrosis-risk as a proxy, and not all patients analyzed here may be appropriate for a MASLD diagnosis. In the cohort, less than 3% were diagnosed for alcoholic liver disease. However, information on alcohol use can only be interpreted modestly in our dataset due to high numbers of missing values. Notably, as our study cohort was composed only of patients with ADRs, we cannot state prevalence of ADRs in patients with high fibrosis risk, but can show poorer outcomes pointing to a clinical relevance of ADRs in this patient group.

Somewhat contrary to our findings, was the observation in our data that the initial triage at ED admission did not show an association of high fibrosis risk with more urgent ADRs nor liver-specific ADR symptoms or admission diagnoses. As the number of patients in these analyses was quite small, the results may differ in larger study populations. However, this may indicate that ADRs do not primary affect the liver or lead to liver failure in patients with high fibrosis risk but can show in a variety of symptoms that nonetheless are connected to a poorer outcome. Since no distinctive differences in adjacent medication were found, the risk of serious outcomes is presumably more related to liver fibrosis-risk and less to drug therapy. In addition, we conducted a general analysis of ADRs without distinguishing whether the drug causing the ADR was metabolized in the liver or not. However, with a median intake of seven to eight drugs, this cohort represents a multi-medicated cohort. Polypharmacy itself can be associated with ADRs and is highly prevalent in patients with chronic liver disease [35]. Alongside with polypharmacy, comes a risk for drug-drug interactions that can increase ADR-toxicity as shown in vitro [36]. Since the expression of drug metabolizing enzymes and drug transporters is expected to be altered under chronic liver disease conditions [37], a modified ADR-risk is likely in these patients. Nevertheless, further analyses are necessary to be able to make a definitive statement. However, further analysis is needed to definitively rule out any influence and to examine a possible cumulative, potential liver-toxic drug effect in more detail. Independent screening programs are therefore particularly important for the early detection of the risk of MASLD-ADR.

The ADRED study is one of the largest studies on ADRs in Germany and therefore provides a good overview of patients who present to the ED due to an ADR. Although patients with minor ADRs who remain undiagnosed or are treated as outpatients are not included, the data from the ADRED study seems representative and holds a broad spectrum of ADR cases and their outcomes. Since the patients in this study did not have a MASLD diagnosis and liver biopsy to confirm fibrosis, the FIB-4 score can only provide an indication of the risk group of patients. Furthermore, surprisingly, there were no significant differences between the two fibrosis risk groups in terms of BMI, diabetes, hypertension and dyslipidemia. However, the nominal prevalence of hypertension and dyslipidemia is higher in the high-risk group, which may indicate a trend, even though it is not statistically significant.

This paper has some limitations. According to the guidelines, the FIB-4 score should only be used for patients diagnosed with NAFLD or MASLD [21]. However, the score can be used to make an initial assessment of potential damage and impaired liver function, as it is also used to assess the risk of other liver diseases of various origins, and its scope of application is constantly expanding [27, 38, 39]. A further limitation is the subjective categorization of the seriousness of ADRs and the discharge status therefore, a dichotomization of the two outcomes was carried out, which enables a clear contrast with precise differentiation.

In conclusion, in patients at risk of MASLD prescribed drugs should be administered if indicated with careful risk assessment, as ADRs with severe progression might occur at any time in the case of undetected liver fibrosis. As there were no distinctive features in the initial assessment in terms of triage category or symptoms even the single use of a non-invasive test may provide important prognostic information in the event of an ADR. Further studies with ADRs in biopsy-confirmed MASLD fibrosis are needed to definitively confirm the results of this paper. Nevertheless, FIB-4 screening of patients presenting to the ED with an acute ADR could identify those at high risk of a serious ADR with a poor outcome.

## Supplementary Information

Below is the link to the electronic supplementary material.

Supplementary Material 1

## Data Availability

The analysed dataset can be accessed upon reasonable request to the corresponding author.

## Declarations

### Clinical trial registration

Registration was carried out via the German Clinical Trials Registry (DRKS-ID: DRKS00008979).
