# Supplementary material for: Association of high risk of liver fibrosis in patients with serious outcomes of adverse drug reactions
Source: Eur J Clin Pharmacol. 2026 Apr 23;82(5):128. doi: 10.1007/s00228-026-04057-z (PMC13102728; doi:10.1007/s00228-026-04057-z)
Supplement: Supplementary file 1 — Supplementary Material 1 [file 228_2026_4057_MOESM1_ESM.docx]

Supplement:

Supplement 1: Outcomes of adverse drug reactions (ADR) in the study cohort (n=1087) stratified by patients with and without liver fibrosis risk according to FIB-4 score

|  | **Missings, n (%)** | **With low or intermediate liver fibrosis risk, n= 680 (62.6%)** | **With high liver fibrosis risk, n= 407 (37.4%)** | **p-value** |
| --- | --- | --- | --- | --- |
| **Triage** | 13 (1.2) |  |  |  |
| Red (Immediate), n (%) |  | 9 (1.3) | 12 (3.0) | >0.05 |
| Orange (very urgent), n (%) |  | 152 (22.5) | 83 (20.8) | >0.05 |
| Yellow (urgent), n (%) |  | 369 (54.7) | 237 (59.4) | >0.05 |
| Green (normal), n (%) |  | 136 (20.1) | 62 (15.5) | >0.05 |
| Blue (non-urgent), n (%) |  | 9 (1.3) | 5 (1.3) | >0.05 |
| **ADR seriousness** | - |  |  |  |
| Not serious, n (%) |  | 2 (0.3) | 1 (0.2) | >0.05 |
| Inpatient treatment, n (%) |  | 635 (93.4) | 350 (86.0) | **<0.05** |
| Intensive care, n (%) |  | 8 (1.2) | 15 (3.7) | **<0.05** |
| Life-threatening, n (%) |  | 34 (5.0) | 34 (8.4) | **<0.05** |
| Death, n (%) |  | 1 (0.1) | 7 (1.7) | **<0.05** |
| **Discharge status** | 42 (3.9) |  |  |  |
| Without damage, n (%) |  | 8 (1.2) | 4 (1.0) | >0.05 |
| Not yet recovered, n (%) |  | 82 (12.6) | 46 (11.6) | >0.05 |
| Condition improved, n (%) |  | 529 (81.5) | 284 (71.7) | **<0.05** |
| Permanent damage, n (%) |  | 5 (0.8) | 4 (1.0) | >0.05 |
| Death, n (%) |  | 25 (3.9) | 58 (14.6) | **<0.05** |

High-fibrosis risk defined as FIB-4 score ≥2.67

Significant findings in **bold** text.

Supplement 2: Most frequently documented drugs in the study cohort (n=1087) stratified by patients with and without liver fibrosis-risk according to FIB-4 score

| **With high liver fibrosis risk, n= 407 (37.4%)** | | **With low or intermediate liver fibrosis risk, n= 680 (62.6%)** | |
| --- | --- | --- | --- |
| Pantoprazole, n (%) | 169 (41.5) | Pantoprazole, n (%) | 249 (36.6) |
| Torasemide, n (%) | 132 (32.4) | Acetylsalicylic acid, n (%) | 185 (27.2) |
| Acetylsalicylic acid, n (%) | 114 (28.0) | Torasemide, n (%) | 173 (25.4) |
| Metoprolol, n (%) | 112 (27.5) | Metoprolol, n (%) | 171 (25.1) |
| Ramipril, n (%) | 81 (19.9) | Ramipril, n (%) | 164 (24.1) |
| Metamizole, n (%) | 81 (19.9) | Metamizole, n (%) | 150 (22.1) |
| Allopurinol, n (%) | 74 (18.2) | Levothyroxine sodium, n (%) | 141 (20.7) |
| Simvastatin, n (%) | 65 (16.0) | Atorvastatin, n (%) | 120 (17.6) |
| Atorvastatin, n (%) | 60 (14.7) | Amlodipine, n (%) | 109 (16.0) |
| Levothyroxine sodium, n (%) | 56 (13.8) | Simvastatin, n (%) | 100 (14.7) |

High-fibrosis risk defined as FIB-4 score ≥2.67
